# Characterization of *Moringa oleifera* Seed Oil for the Development of a Biopackage Applied to Maintain the Quality of Turkey Ham

**DOI:** 10.3390/polym16010132

**Published:** 2023-12-30

**Authors:** Lesly Adamari Cueto Covarrubias, Mónica Andrea Valdez Solana, Claudia Avitia Domínguez, Alfredo Téllez Valencia, Jorge Armando Meza Velázquez, Erick Sierra Campos

**Affiliations:** 1 Facultad de Ciencias Químicas GP, Universidad Juárez del Estado de Durango, Av. Artículo 123 S/N Fracc. Filadelfia, Gómez Palacio 35015, Durango, Mexico; 2 Facultad de Medicina y Nutrición, Universidad Juárez del Estado de Durango, Av. Universidad y Fanny Anitúa S/N, Durango 34000, Durango, Mexico

**Keywords:** *M. oleifera* seed oil, biopolymers, biopackage, ham preservation

## Abstract

*Moringa oleifera* has a high level of active chemicals that are useful in the food industry, and they have antibacterial and food preservation properties. The characterization of *M. oleifera* seed oil (MOS) may vary due to agronomic and environmental factors. Therefore, it was necessary to know the composition of lipids present in our oil extracted under pressing at 180 °C and thus determine if it is suitable to produce a biopackaging. Within the characterization of the oil, it was obtained that MOS presented high-quality fatty acids (71% oleic acid) with low values of acidity (0.71 mg KOH/g) and peroxide (1.74 meq O_2_/kg). Furthermore, MOS was not very sensitive to lipoperoxidation by tert-butyl hydroperoxide (tBuOOH) and its phenolic components, oleic acid and tocopherols, allowed MOS to present a recovery of 70% after 30 min of treatment. Subsequently, a biopackaging was developed using a multiple emulsion containing corn starch/carboxymethylcellulose/glycerol/MOS, which presented good mechanical properties (strength and flexibility), transparency, and a barrier that prevents the transfer of UV light by 30% and UV-C by 98%, as well as a flux with the atmosphere of 5.12 × 10^−8^ g/ m.s. Pa that prevents moisture loss and protects the turkey ham from O_2_. Hence, the turkey ham suffered less weight loss and less hardness due to its preservation in the biopackaging.

## 1. Introduction

Plastics play a critical role in food packaging, keeping food quality and safe, ensuring acceptable shelf life, and helping to reduce food waste. However, the consumption of these materials is a significant contributor to environmental challenges such as resource depletion, trash, and global warming [1]. Furthermore, with greater public awareness of the environmental issues associated with traditional plastic materials, it is therefore very necessary to replace non-degradable petrochemical packaging with edible or biodegradable materials [2,3].

Biodegradable polymers are a viable alternative to traditional non-biodegradable plastics when recycling is not feasible or economical [4]. Compostable food packaging has the potential to solve the disposal issues of plastic packaging that is difficult to recycle, thereby preventing it from being disposed of in landfills or incinerated. Biopolymers might be proteins, polysaccharides, lipids, or a combination of these. Biopackaging applications have been suggested to use polysaccharides like cellulose, alginate, and gelatin as biopolymeric materials [5]. Hence, compostable food packaging can improve the quality of organic waste, which can be used to restore soil nutrition by properly treating it [6]. However, there are some disadvantages to using lignocellulosic materials as reinforcing agents, such as their ability to absorb high humidity, poor wetting ability, and incompatibility with most biopolymers [7]. While glycerol has various desirable properties, including being affordable, food-grade, biodegradable, heat-resistant, and non-toxic. Glycerol (Gly) has also been shown to improve cellulose-based film elongation at break, flexibility, and biodegradability [8]. These compounds may also be used to encapsulate antimicrobials, antioxidants, probiotics, and flavoring chemicals, therefore protecting food, extending shelf life, and improving the qualities of components such as appearance, sensory attributes, and freshness. For example, whey protein isolate nanofibers (WPNFs) were recently combined with anthocyanin (ACN), Gly, and the polysaccharide pullulan (PU) to form the intelligent food packaging (WPNFs-PU-ACN/Gly). ACN and Gly were used as the color indicator and plasticizer, respectively, while PU was added to improve the mechanical properties of the WPNFs-PU-ACN/Gly edible film. As a result, because the color shift of ACN was perfectly compatible with fish pH, this film would be excellent for sensing the pH of salmon, which changes with degradation [9].

Fresh poultry and pork meat products represent highly perishable products that are susceptible to spoil within a few days after production. Spoilage of meat products is mainly due to microbial growth and chemical defects such as lipid and protein oxidation. It results in sensory alterations with the change of color or texture and the development of off-odors, slime, etc. [10]. Processing steps such as mincing, cooking, and salt addition that promote the formation of reactive oxygen species (ROS) increase the susceptibility of products to oxidation. It is reported that lipid oxidation, being a major cause of off-flavor and discoloration in meat and meat products, can result in a product that is undesirable for human consumption, and ROS-induced protein oxidation can result in modifications to the backbones and side chains of proteins, leading to structural changes at the levels of primary, secondary, and tertiary structures of proteins [11].

Numerous research works have suggested that the lipid and protein oxidation in processed meat products can be retarded or inhibited by the supplementation of commercial synthetic or natural antioxidants. Treatments with antioxidants resulted in significantly lower peroxides, conjugated dienes, and thiobarbituric acid-reactive substances and were also effective in retarding the formation of carbonyl groups, reducing the loss of sulfhydryl groups, and protecting α-helix contents, of which ferulic acid and rutin were the most effective [12]. Therefore, incorporating potential bioactive compounds into biodegradable packaging seems to be a promising technique for resolving this issue, providing a sustainable alternative to traditional packaging, and extending the shelf-life of food. For this reason, we consider that the *Moringa oleifera* would be a source of suitable additives for food packaging materials.

*Moringa oleifera* possesses an interesting antioxidant activity and acts as a good source of antioxidants due to the presence of several types of compounds, such as ascorbic acid, flavonoids, phenolics, and carotenoids [13]. Phenols play an important role in reducing fat oxidation, inhibiting protein degradation, and increasing fat stability and the shelf-life of chicken burgers, which are highly consumed products [14]. Strong inhibitory effects of *Moringa oleifera* seed kernel extract were observed for *Bacillus cereus*, *Staphylococcus aureus*, *Mucor species*, *and Aspergillus species* [15]. According to a recent study, only apolar extract obtained from *M. oleifera* seeds displayed anti-microbial activity against Gram-positive bacteria [16]. Therefore, *M. oleifera* seed oil (MOS) may be an attractive component for biodegradable food packaging owing to its natural origin and functionality (antimicrobial/antioxidant activity). Thus, the use of *Moringa oleifera* oil in food packaging was explored due to its high concentration of bioactive components, which may improve the functional qualities of packaging systems and lengthen food shelf-life. Despite a large body of literature on the extraction optimization and chemical characterization of active compounds from *M. oleifera* oil, their impact on the functional qualities of food packaging materials remains unknown. Thus, the primary goals of this research are to identify the main bioactive components in *M. oleifera* seed oil, to evaluate the major advances in their extraction, purification, and characterization, and lastly, to apply them in food packaging systems. This study aims to assess the ability of new packaging to preserve the quality of turkey ham in comparison to classic polyethylene terephthalate packaging.

## 2. Materials and Methods

### 2.1. M. oleifera Seed Oil Extraction

*M. oleifera* seeds were collected from Mexican cultivar (Lombardia, Michoacán, located on 19°01′30″ N and 102°05′39″ W, Mexico, April 2023). The seeds of *M. oleifera* were hulled, and the integument was pressed at 180 °C. During the process, the seeds are ground, obtaining oil and a residue of the defatted seed, which is pressed in an analogous way three times continuously. The oil obtained was centrifuged at 3500 rpm for 5 min, to eliminate the fine particles still present in suspension in the oil. Finally, the oil free of impurities was stored at room temperature (25 ± 3 °C) for subsequent analysis [17,18].

### 2.2. Physico-Chemical Characterization of the MOS

The quality of edible oil composition can be evaluated using various physical and chemical parameters [19,20]. Iodic value (IV), saponification value (SV), viscosity, peroxide value (PV), and acidity values (AI) are some of the main physicochemical parameters evaluated. The methods used for each were based on the AOAC [21], except for the iodine value, which was determined by the Rosenmund–Kuhnhenn method [22]. 

#### Antioxidant Capacity and Total Phenols in MOS

The free radical scavenging activity of MOS was evaluated against 1,1-diphenyl-2-picrylhydrazyl DPPH (0.83 mM) and 2,2’-Azino-bis(3-ethylbenzothiazoline-6-sulfonic acid) (ABTS^•+^) (7 mM). A volume of 10 µL of MOS was added to 590 µL of the methanol solution of DPPH or ABTS. The absorbance was read against the reagent blank at 510 nm and for ABTS at 730 nm with an incubation time of 30 min. The free radical inhibition by DPPH and ABTS was calculated using Equation (1) [23,24]. Finally, the content of total phenols in MOS was determined using the Folin-Ciocalteu reagent. For this, a mixture of 10 µL of MOS, 160 µL 7.5% Na_2_CO_3_, and 260 µL of 0.2 M Folin-Ciocalteu was made. Subsequently, it was left to incubate for 20 min at room temperature and then read at 760 nm. The amount of total phenols in MOS was determined from the trolox calibration curve and expressed as mg trolox equivalent per gram of sample (mg trolox g^−1^) [21].

(1) $$I\%=\frac{\left(A_{\mathrm{control}}-A_{\mathrm{sample}}\right)}{A_{\mathrm{control}}}*100$$

A_control_ = absorbance of the reagent (DPPH or ABTS), A_sample_ = absorbance of MOS after 30 min incubation

### 2.3. Fatty Acid Profile of MOS

One µL of the methylated ester extract (FAME) was injected into an Agilent 6820 Ga-ses chromatograph (Agilent Technology, Santa Clara, CA, USA) equipped with an ionization flame detector. The FAME were separated on a Supelco SP-2560 capillary column (120 m × 0.25 mm × 0.2 µm) using helium as carrier gas at a pressure of 50 psi. The operating conditions of the chromatograph were as follows: injector in splitless mode at an initial temperature of 110 °C with an increase of 5 °C/min until 225 °C was reached. High purity standards of FAME were used not only to identify but also to quantify these compounds (R^2^ ≥ 0.99). Fatty acids were expressed as a percentage of the total content. The sample was saponified with sodium methoxide and esterified with boron trifluoride.

### 2.4. MOS Susceptibility to Oxidative Stress

The evaluation of the redox state of MOS was carried out by spectrophotometric analysis with a scanning approach between 200 and 350 nm. For the development of the evaluation, the spectral signal of MOS, which was dissolved in cyclohexane (1:140), was considered. Subsequently, two concentrations (87 µM and 1.72 mM) of the oxidising agent tert-butyl hydroperoxide (tBuOOH) were added. The mixtures were incubated for 30 min and read at 5 min intervals. Trolox (172 µM), an antioxidant analog of vitamin E, was used as a standard control. It was submitted to the same conditions as MOS. For the interpretation of the results, the changes in the spectral signal were analysed concerning the incubation time [25,26].

### 2.5. Development of a Biopackaging with MOS

To prepare the biopackaging emulsion, 1.2% of carboxymethylcellulose was dissolved in 175 mL of deionized water under constant stirring at a temperature of 70 °C. Once the solution was dissolved, 2% of corn starch, previously dissolved in 25 mL of deionized water, was incorporated. The resulting solution was kept under constant stirring until reaching a temperature of 90 °C to activate the starch. Subsequently, the temperature was slowly decreased until reaching 42 °C to add glycerol (1%) and MOS (0.75%), stirring for another 5 min to achieve a homogeneous mixture and pouring it onto a glass mold. Drying was carried out in an incubator at 40 °C for 24 h, ensuring dispersion and uniformity of the biopackaging before drying. 

### 2.6. Opacity and Filters of Biopackaging

The biopackage was cut into a rectangular piece and placed in a quartz cell for spectrophotometric analysis, recording its absorbance at 600 nm. The thickness was measured with a digital micrometer (QuantuMike) and the mean value was determined by averaging the thickness at different positions. Subsequently, the opacity of the biopackaging was calculated using Equation (2) [27].

(2) $$\mathrm{Opacity}=\frac{A_{600}}{I}$$

A = absorbance of the film at a wavelength of 600 nm, I = Film thickness (mm).

The amount of UV light passing through the biopackaging was assessed by spectrophotometric analysis. In the same way as the previous evaluation, the biopackage was cut into a rectangular piece and placed inside the quartz cell. The maximum absorbance value was taken as a reference but for each type of ultraviolet light; UV-C corresponds to 200 to 280 nm, while UV-B ranges from 280 to 315 nm and UV-A from 315 to 400 nm, respectively. For the interpretation of the results, it was necessary to perform the transformation from absorbance to transmittance [28].

### 2.7. Water Vapor Permeability (PVA) of Biopackaging

In the evaluation of this parameter, the standard test methods for water vapor transmission (ASTM E96-00e1) [29] were considered, but with some modifications. In a beaker, 20 mL of water was added, and a part of the biopackaging was placed on the top of the beaker in a circular shape so that it remained adhered to the beaker. It was then placed in a desiccator with a hygrometer and silica gel to keep the humidity under control for 24 h. During the incubation time, the weight was recorded at 30-min intervals. Water vapor permeability was expressed in g/m* s*Pa and calculated using Equation (3) [30].

(3) $$\mathrm{PVA}=\frac{∆m-I}{A-∆t-∆P}$$

∆m = grams lost per second (g), I = thickness of biopackage (m), A = area of exposed biopackage (m^2^), ∆t = unit of time (1 s), ∆P = pressure (Pa).

In addition, Equation (4) was used to calculate the pressure variation between the faces of the biopack and the environment.

(4) $$∆P=\frac{∆\mathrm{RH}}{100}*\mathrm{PVAP}$$

∆RH = humidity percentage between ambient (36%) and inside the vessel (100%), PVAP = water vapor pressure saturation equivalent to 3160 Pa

### 2.8. Protective Capacity of Biopackaging on the Useful Life of Turkey Ham

The ham slices were placed in the biopackaging and stored at 4 °C. Samples were then taken at different times (4, 17 and 21 days) to evaluate their weight loss based on their moisture content, the hardness of the ham with respect to the force required to make a cut, the color (L*, a*, b* scales) were collected with a CR-400 colorimeter (KONICA MINOLTA, USA). Delta *E* measures the magnitude of difference in L*, a*, and b* color space. Delta *E* was calculated for all frozen storage periods using the initial color readings taken during fabrication and the final readings when steaks were removed from the freezer in the frozen state for each individual steak. Delta *E* was then calculated using the equation ∆*E* = [(∆L*)^2^ + (∆a*)^2^ + (∆b*)^2^]^1/2^ from the values obtained by the colorimeter [31].

### 2.9. Statistical Analysis

Experimental data were expressed as the mean ± SD of the triplicate trial. A three-way analysis of variance (ANOVA) was estimated to determine if there is a statistically significant difference between the means of the treatments (control, biopackage and synthetic packaging), applying the Tukey multiple comparisons test (*p* ≤ 0.05). All statistical correlation calculations were processed with Medcalc version 12.3.

## 3. Results and Discussion

### 3.1. Physico-Chemical Composition of M. oleifera Seed Oil

Several researchers studied the impact of temperature on the stability of their properties to assess their quality and functionality [32,33,34]. Their research highlights that high temperatures affect stability, mainly in AI parameters and PV. 

Table 1 shows the physicochemical parameters of MOS, extracted by pressing at 180 °C, a crucial factor that can influence the quality and stability of the oil [35]. Despite the above-mentioned studies, the AI in MOS was 0.71 mg KOH/g, indicating that the triglycerides were not hydrolyzed. Also, the PV is a measure of the degree to which rancidity reactions have occurred during storage; in MOS, it was 1.74 meq O_2_/kg, corroborating that no significant oxidation of unsaturated fatty acids occurs in our sample. As for the following parameters, SV is an index of the average molecular mass of the fatty acid in the oil sample and the value in MOS was 184.06 ± 0.427 mg KOH g^−1^, confirming the presence of long-chain and/or unsaturated fatty acids with a high molecular weight and a high melting point. To confirm the above values, the IV parameter indicates the degree of establishment of a vegetable fat or oil. This determines the stability of the oils against oxidation and allows a qualitative determination of the overall setting of the fat [36,37]. MOS had a very low IV (30.96 ± 0.707 I_2_ 100 g^−1^), showing a higher establishment in its fatty acid chain.

Table 1 also presents the parameters of viscosity, color, and antioxidant capacity. Oils with lower viscosity values are highly appreciated by consumers. Their viscosity depends on the nature of the TG present in the oil because the fatty acids in the glycerol backbone of the triglyceride molecule cause a change in viscosity. Therefore, the MOS value obtained was related to chain length and saturation/unsaturation. Similarly, color is a crucial indicator to assess the quality of an oil. Color alterations in oils can be due to the production area, the extraction process, and the preservation of the oil. MOS is characterized by faint yellow tones with intense green highlights, as indicated by the parameters (L, a, and b, see Table 1). These results agree with another study suggesting how the extraction process affects oil color by comparing the color values with enzymatic extraction (0.7R + 5.9Y) and solvent extraction (0.7R + 3.0Y). The R values were not altered, retaining their yellow-red colors, but the Y value is affected by the extraction method, indicating a decrease in lightness, possibly due to oxidation of the oil [38].

The antioxidant capacity of an oil is an important parameter that plays an active role in many fields, due to the preservation and stability it can provide to food components. Table 1 shows the % inhibition per 10 µL of MOS, as well as its total phenol content. The antioxidant capacity present in MOS does not show significant values. However, other studies obtained values of 17% for DPPH and 28% for ABTS; they also corroborated that, with an amount greater than 50 µL, it can reach inhibition values of 60 ± 10% [21,39]. Therefore, the presence of total phenols in MOS allows it to have an antioxidant capacity, which makes it a potential alternative as a natural antioxidant.

### 3.2. MOS Fatty Acid Profile

Phenolic compounds and the nutritional quality of the fatty acid profile can be affected by environmental, harvesting and seed processing conditions, an essential factor in determining the potential applications of the oil [40]. Based on the high degree of unsaturation mentioned above, it was decided to know its fatty acid profile using gas chromatography. The MOS presented a high degree of monounsaturated fatty acids (MUFA) at 75%, highlighting oleic, palmitoleic, and eicosene. However, the percentage of polyunsaturated fatty acids (PUFA) was minimal, highlighting only the presence of licnoleic, and the main saturated fatty acids were behenic, stearic, and palmitic (Table 2).

MOS contains all the major fatty acids found in olive oil [41], therefore, it can be used as a possible substitute for expensive olive oil after some modifications. Based on the comparison of its fatty acid profile, it shows the presence of behenic acid (C22:0), lignoceric acid (C24:0) and traces of n-pentadecanoic and pentadecenoic acid [42,43]. In addition, the extraction method is a determining factor in its fatty acid composition, a solvent and enzyme-extracted oil from *M. oleifera* seed [38] showed that solvent-extracted oil has 67.9% oleic acid compared to 70.0% for enzyme-extracted oil, palmitic (7.8% and 6.8%), stearic (7.6% and 6.5%) and behenic (6.2% and 5.8%), respectively.

### 3.3. Susceptibility of MOS to Oxidative Stress

Controlling lipid oxidation during processing and storage is critical for food quality. Lipids can slowly oxidize due to a variety of environmental factors such as oxygen in the air, light, temperature, and metal ions. This results in a continuous chain reaction of free radicals, which produces hydroperoxides and secondary chemicals such as aldehydes and ketones, resulting in a rancid product with diminished flavor, color, and nutritional value [44,45]. Spectroscopic methods are efficient tools for food in terms of oxidative stability and quality because they are highly sensitive, quick, and can be performed directly on samples without any prior treatments [25]. UV-Vis absorption spectroscopy is a technique that detects single pigment contents in the visible range (400 nm to 800 nm) and other compounds present in olive oil in the UV range (230 nm to 400 nm), such as polyphenols, peroxides, and other fatty acid derivatives due to oil oxidation [46,47].

**Figure 1:**
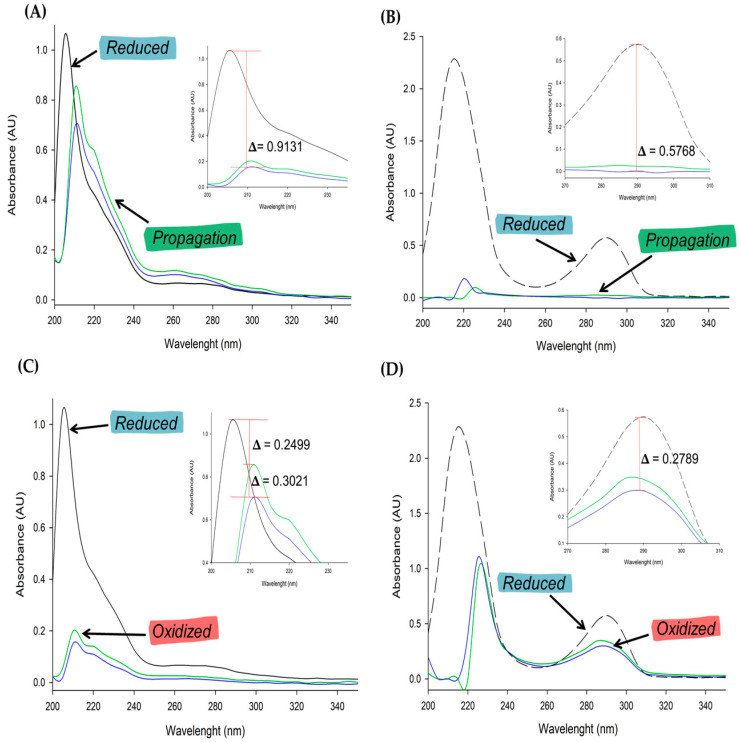
Absorption spectra in the UV region for MOS (**A**) initial reaction (1 min) between MOS (black line) with 87 µM (green line) and 1.72 mM (blue line) of tBuOOH (**C**) after reaction (30 min) with tBuOOH. Absorption spectra in the UV region for Trolox (**B**) initial reaction (1 min) between trolox (black line) with 87 µM (green line) and 1.72 mM (blue line) of tBuOOH (**D**) after reaction (30 min) with tBuOOH.

UV-visible spectroscopy is being used to determine whether MOS becomes more sensitive to oxidative stress when tBuOOH is added, as well as whether phenolic compounds found in MOS act as antioxidants. Figure 1A depicts the absorbance spectrum of MOS in the UV region, with a high peak between 200 and 220 nm, indicating the presence of phenols. There is also a shoulder between 200 and 240 nm and a slight peak between 260 and 280 nm. These results agreed that the absorption at 236 nm may be attributed to different compounds present in the vegetable oil, such as phytocholesterols [48], and the absorption band at 293 can be attributable to tocopherols and FAs [49]. The above-mentioned spectrum (green line) shows a significant reduction after the addition of 87 µM tBuOOH. The addition of 20 times more tBuOOH has no effect on the oxidized spectrum of MOS, indicating that MOS is highly susceptible to the oxidizing agent at the start of the reaction. Finally, the internal plot in Figure 1A shows the delta parameter, which represents the absorbance difference between the oxidized (blue line) and reduced (black line) spectra, with a value of 0.9131.

In addition, to validate the usefulness of the UV spectral method using trolox as a control antioxidant standard, Figure 1B shows the spectral signal of trolox in its reduced form, presenting two peaks, one in the region of 200–240 nm and the other between 280 and 300 nm (black line). In comparison to MOS, the addition of 87 M of tBuOOH results in an even greater spectrum reduction (blue line). The parameter ∆ was 2 at the first peak. It completely reduced in the second peak, and the delta value between its reduced and oxidized spectrums was 0.5768 (internal plot). 

The UV spectrum of the MOS and trolox were obtained again after 30 min of incubation with the oxidizing agent, yielding very interesting results. The signal increased significantly during MOS strokes, and a similar pattern was observed between the 200 and 240 nm ranges. In Figure 1C, the signal with 87 M tBuOOH (green line) added showed significant changes compared to Figure 1A, with a value of 0.2499. The delta value was 0.3021 for a concentration of 1.72 mM (blue line). There was an increase in the signal of both peaks in trolox (Figure 1D). The peak between 280 and 310 nm shifted to a value of 0.2789 (blue line). 

Resistance to the generation of harmful compounds during lipoxidation is due to the components present in MOS, phenols, α-tocopherols, and oleic acid. Oils such as olive oil can regenerate their antioxidant molecules, achieving rapid repair of oxidized lipids [50]. Furthermore, the presence of phenols and oleic acid modulates the degree of oxidation [51]. After 30 min, the antioxidant molecules regenerated from MOS neutralized the lipid radicals formed by H^+^ donation. As a result, the production of stable compounds increases their signal. Trolox is similarly converted to its stable form as hydroquinone trolox, with a recovery like MOS. It should be noted that the recovery rate in MOS was 70–60%, whereas the recovery rate in trolox was 40.29–48.31% (Panels C and D). These results showed the ability of UV-vis spectrometry to characterize the amount of oxidation products in MOS using oxidative stress simulation.

### 3.4. Opacity and Filters of the Biopackaging

Light energy in the ultraviolet regions, moisture, gas exchange and the presence of microorganisms play a major role in the overall quality of food [52]. Transparency is the physical property of objects that allows light to pass through them without scattering. For consumers, it is the most valuable characteristic, as it allows them to assess the freshness and appearance of the product [53]. This parameter allows for the understanding of the effect that UV light has on the biopackaging barrier, which is important when evaluating and validating a packaging material.

During the spectrophotometric measurement the light must pass through the amorphous/crystalline/polymeric interfaces. Through this the biopackage presented an opacity of 0.0675 Abs600/mm. Therefore, the UV-C light captured by the biopackaging was 1.71% and for UV-B and UV-A it was captured between 65 and 70.16%. Consequently, the biopackaging is classified as translucent (ranging from 10 to 80%) and opaque (<10%), although it does not fall within the threshold to be considered transparent, the biopackaging allows UV-B and UV-A blocking of 30% and 98.3% for UV-C, thus falling within a range of commercial interest for certain food storage. In general, the opacity in a PET material prevents the passage of radiation at wavelengths below 340 nm, obtaining a transmittance between 60 and 70% [28,54]. Although there are also PET materials with values between 30 ± 2% [55]. Taking this into account, biopackaging with the addition of MOS allows it to have barrier properties like a PET material, considering it as a protective packaging for photosensitive foods.

### 3.5. Water Vapor Permeability of Biopackaging

Water vapor permeability (WVP) is a crucial property to assess the barrier capacity of a material, mainly because of the role of water in food spoilage reactions, as it leads to alterations in shape and texture, causing dehydration and loss of compounds in the food [56]. Several factors can affect permeability, such as temperature, thickness and plasticizer content [57]. In biopackaging, its WVP value was 5.12 × 10^−8^ g/m.s.Pa, which allows slow vapor transfer between the walls of the material, resulting in a controlled transport of water vapor and mass, therefore can prolong the shelf life of the products.

In the development of a biofilm from cellulose, they obtain a WVP of 1.7 × 10^−4^ g/m.s.Pa, which indicates a higher moisture transfer [58]. On the other hand, the WVP in a PET material is found to be 3.62 × 10^−7^ g/m.s.Pa [55]. In other words, the use of carboxymethyl cellulose in biopackaging has a negative effect on its barrier properties, favoring moisture transport and potentially affecting the product. Therefore, compounds such as corn starch, glycerol, and MOS can lead to more stable values, allowing values closer to those of a PET material.

### 3.6. Protective Capacity of Biopackaging on the Useful Life of Turkey Ham

Turkey ham is a processed meat product made by mixing cooked or cured turkey meat and water. It is a highly perishable food that relies on refrigeration and suitable packaging to protect it from biochemical or mechanical damage, thus prolonging its shelf life. Lipid and pigment oxidation are the main causes of quality degradation in ham, causing discoloration, drip loss, development of bad odors, creation of potentially toxic compounds and changes in nutritional characteristics [59,60,61]. The use of biopackaging based on biopolymers and MOS is a possible alternative to synthetic packaging to extend the shelf life of turkey ham, allowing the preservation of its properties.

**Figure 2:**
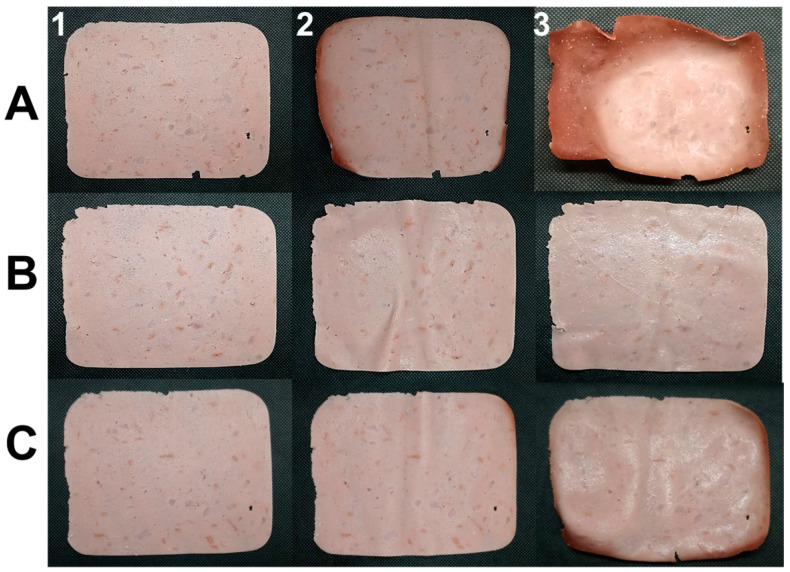
Storage of Ham. Tratments: (**A**) control (**B**) Synthetic package (**C**) Biopackage. Time: (**1**) Day four (**2**) Day seven (**3**) Day twenty-two.

Figure 2 illustrates the storage of each treatment from day 4 to day 27. The control sample experienced changes in color, shape, and texture (Panel A), these significant changes occurred from day 7 due to direct exposure to light, temperature, and oxygen. On the other hand, when comparing the biopackaging treatments with the synthetic polyethylene terephthalate container (Panel B with C), the changes were minimally significant, since the biopackaging exerted a protective function against the factors mentioned above in the control. Therefore, it was able to retain its properties for a longer period of time, however, from day 27 it started to show colour changes (Panel C, 3). In contrast, the synthetic packaging did not show any changes (Panel B, 3), so this type of material protects its properties for a longer period of time.

Table 3 shows the variations in humidity over time in each of the treatments. The initial humidity of the ham was 76%, which varied significantly between days 4 and 7. However, on day 22, the humidity percentage in the control sample decreased to 27.45%. In the case of biopackaging and synthetic packaging, the percentage of humidity was similar for days 4 and 7 but there was only a loss of 8 and 6%, respectively, for day 22. Therefore, our findings show that biopackaging was as effective as synthetic packaging at reducing moisture loss.

The discharge of water induced dehydration in the turkey ham, which entirely changed its texture and increased its hardness. For this reason, we assess the texture of each treatment at various storage durations. On day 4, a cut force of 1.95 was necessary for the control treatment, and this force was increased three times on day 7. Finally, on day 22, a force of 122.603 ± 0.9211 N was required, which was 62 times greater than the force necessary on day 4. In contrast, the same cut force was required for each of the storage durations for the biopackaging and synthetic, which was 90 times lower than the control for day 22 (Table 4).

Previous studies had confirmed that light had a detrimental effect on the color stability of cooked ham. Products exposed to light showed higher values of L*, metmyoglobin (Mb%) and hue angle, and lower values of a*, a*/b*, Chroma and nitrosomyoglobin [62]. 

According to Table 5, the control sample with twenty-two days of storage time at 4 ± 2 °C had the highest L* value, while the control with four days of storage time had the lowest L* value. These findings are the result of an increase in moisture loss. Furthermore, the greatest a* and b* values for control belong to days 4 and 7, respectively. The luminosity in the control sample increased from day 4 to day 7 and this value was maintained until day 22. In contrast, the value of parameter a* underwent a change from 7.34 for the fourth day to −3.35 for day 22, which represents a change from red to green. Finally, the parameter b* in the control went from −8.01 for the fourth day to 3.52 for the seventh day and this value decreased slightly for day 22 to 2.14, which represents a change from blue to yellow. In contrast, the biopackaging sample showed a smaller change in luminosity than the control (Table 5) and its parameters a* remained at positive values with a significant decrease between day 4 and 22. While the parameter b* remained at negative values with a significant difference between day 4 and the seventh day. It is important to mention that the color parameters for the biopackaging were like the results of the synthetic packaging and there were no significant differences between these treatments for each of the parameters evaluated (Table 5). Also, the data obtained show that there is a clear visible total difference (∆E) in the ham slices of the control and biopacking and synthetic packaging (Table 5). The values of ∆E obtained for control sample, biopackage, and synthetic packaging were 22.37, 8.1, and 7.37, respectively. According to some authors, differences in color can be seen by the human eye at a quick glance at values of ∆E from 2 to 10 [63]. Therefore, all treatments undergo apparent color changes at day 22 that could be noticeable by the consumer. 

## 4. Conclusions

Various natural preservatives, such as essential oils or other phytochemical extracts, may be incorporated to improve the efficacy of the biopolymer network. This procedure is very beneficial because it extends the shelf life and preserves packaged items. MOS’s bioactive components (phenols, oleic acid, and tocopherols) provide it with anti-oxidant capacity, allowing it to be highly resistant to autoxidation and tolerate both high temperatures and oxidizing chemicals. As a result, this was used to create a bioopackaging that effectively stopped weight, color, and hardness loss in turkey ham during storage. MOS’s antioxidant capability confers barrier and mechanical qualities, making it potentially viable as a replacement for polyethylene terephthalate packaging. This study, however, did not include any microbiological testing. As a result, a study to understand how the antioxidant capacity of MOS affects biopackaging to limit bacterial and fungal growth in meat sausages is advised.

## Figures and Tables

**Table 1:** Results of physicochemical properties of MOS.

Parameter	Value
Acidity	0.71 mg KOH/g
Peroxide	1.74 meq O_2_/kg
Saponification	184.06 ± 0.427 mg KOH g^−1^
Iodine	30.96 ± 0.707 I_2_ 100 g^−1^
Viscosity	51.5 MPa·s (20 °C)
L*	96.143 ± 5.889
a*	−6.993 ± 1.810
b*	22.953 ± 1.716
DPPH	17.30 ± 0.636%
ABTS	11.89 ± 0.784%
F-C	8.58 ± 0.235 mg trolox g^−1^

Mean, ± corresponds to the standard deviation.

**Table 2:** Fatty acid profile of MOS.

Fatty Acids	Percentage
Palmitic (C 16:0)	5.45
Palmitoleic (C 16:1)	1.64
Estearic (C 18:0 C)	7.03
Oleic (C 18:1 w-9)	71.7
Linoleic (C 18:2 w-6)	0.54
Araquidic (C 20:0)	4.21
Eicosene (C 20:1 w-9)	1.67
Behenic (C 22:0)	6.93
Lignoceric (C 24:0)	0.84
SAFA	24.46
MUFA	75.01
PUFA	0.54

(SAFA) Saturated Fatty Acid, (MUFA), Monounsaturated Fatty Acid, (PUFA) Polyunsaturated Fatty Acid, (w-9) omega 9, (w-6) omega 6.

**Table 3:** Moisture determination in ham.

Time	Control	Biopackage	Synthetic Package (PT)
Four	74.16 ± 0.86% ^a^	74.16 ± 0.86% ^a^	74.16 ± 0.86% ^a^
Seven	71.36 ± 1.05% ^a^	72.52 ± 0.31% ^a^	72. 96 ± 1.52% ^a^
Twenty-two	27.45 ± 1.94% ^b^	68.47 ± 0.21% ^b^	70.02 ± 1.14% ^ab^

Mean, ± corresponds to the standard deviation. Time is expressed in days. Polyethylene terephthalate (PT). The letters represent significant changes between different days of storage (*p* < 0.05).

**Table 4:** Evaluation of texture of turkey ham in the different storage.

Time	Control	Biopackage	Synthetic Package (PT)
Four	1.9583 ± 0.2574 N ^a^	2.2555 ± 0.1995 N ^a^	2.1755 ± 0.1218 N ^a^
Seven	6.8641 ± 0.9726 N ^b^	4.1623 ± 0.129 N ^b^	4.5923 ± 0.096 N ^b^
Twenty-two	122.603 ± 0.9211 N ^c^	4.0761 ± 0.3344 N ^b^	4.0179 ± 0.1717 N ^b^

Mean, ± corresponds to the standard deviation. Time is expressed in days. Polyethylene tereph-thalate (PT). The letters represent significant changes between different days of storage (*p* < 0.05).

**Table 5:** Color determination in ham.

	Control				Biopackage				Synthetic Package (PT)			
Time	L*	a*	b*	∆E	L*	a*	b*	∆E	L*	a*	b*	∆E
Four	74.54 ± 2.13	7.34 ± 1.96	−8.01 ± 1.53		73.8± 0.25^a^	5.34 ± 1.33 ^a^	−5.01 ± 1.87 ^a^		75.6± 0.44 ^a^	6.77 ± 1.45 ^a^	−7.66 ± 0.90 ^a^	
Seven	91.11 ± 2.14 ^b^	−2.2 ± 0.92 ^b^	3.525 ± 0.94 ^b^		79.3 ± 2.96 ^b^	3.53 ± 0.59 ^b^	−2.84 ± 2.72 ^b^		76.8 ± 1.09 ^a^	5.53 ± 0.11 ^b^	−4.66 ± 1.41 ^b^	
Twenty-two	91.37± 1.81 ^b^	−3.35 ± 1.66 ^ab^	2.14 ± 0.86 ^ab^	22.37 ± 1.03 ^b^	81.7± 1.34 ^b^	4.69 ± 0.83 ^b^	−5.28 ± 2.84 ^ab^	8.1 ± 0.34 ^b^	79.1± 1.40 ^b^	4.43 ± 1.11 ^b^	−3.03 ± 0.71 ^ab^	7.37± 1.04 ^c^

Mean, ± corresponds to the standard deviation. Time is expressed in days. Polyethylene terephthalate (PT). The letters represent significant changes between different days of storage (*p* < 0.05). Visual discoloration was based on a scoring system of 0–100, where zero is 0% surface discoloration.

## Data Availability

The data presented in this study are available on request from the corresponding author.
